# Monthly Summary

**Published:** 1885-06

**Authors:** 


### MONTHLY SUMMARY.

DANGERS OF WEARING SMALL DENTURES.—*By John Trude Fripp, L. D. S. R. C. S. I.*—In the *British Journal of Dental Science*, for the first of February, a case is recorded in illustration of the danger of sleeping with artificial teeth. From the fact that the plate passed safely through the alimentary canal I should judge it must have been a very small one. Certainly patients should be warned of the danger of keeping an artifical denture in the mouth at night; but I would go a step further, and urge that there is considerable danger incurred in wearing very small dentures at any time. A lady came to me only a few months ago with a small vulcanite plate, carrying the two upper bicuspid teeth of the right side, which she told me she had swallowed, but happily passed safely per rectum. They had become displaced during dinner, and passed into the oesophogus with the food before she became aware of what had happened. I had warned her previously of the danger, as the attachments were not secure, but the plate had been worn for several years and the danger was not appreciated until the accident occurred. It may, of course, be urged that had the dentures in these two cases not been so small the wearers could not have passed so happily through the accidents, but on the other hand is it not reasonable to urge that had they been *much* larger the possibilities of their being swallowed at all would have been much reduced.

I have for some time given up the making of small cases, and even for a single front tooth prefer to put in a palate.

A small case always requires clasps, which in the larger cases may very often be dispensed with.

There are certainly three distinct advantages in making the cases larger. (1.) Much greater firmness is ensured. (2.) The natural teeth are not so likely to be injured as by the tight clasping of bands and wires. (3.) And there is much less likelihood of a case being swallowed.—*Dental Advertiser.*

---

AN APPETITE FOR MORTAR.—The following peculiar case seems to possess sufficient interest to warrant reproduction. It is reported in the *Lancet*, January 31, 1885, by Dr. Charles E. Adams:

W. T——, aged three years, is a pale, unhealthy looking boy, decidedly rickety, with thickened bones at wrist and ankle joints, carious teeth, and enlarged abdomen; he is also backward in his walking, not having full locomotive powers. His mother informed me that up to two years of age he was a fine baby; he then had an attack of bronchitis, and was treated at a London hospitial, where he was offered admission, which his mother refused. The attack left him in a weak state, and soon after that time he showed signs of rickets. About eight months ago he exhibited a great desire for eating mortar. The mother discovered his propensity by observing that the wall near his bed was stripped of paper and holes picked in it. On inquiring into the cause, she found that the boy used to eat the mortar, and his eagerness after it was so great that he would get, even in inclement weather, into the yard and pick the walls, and if prevented he cried. On occassions the child has been deprived of his mortar, and invariably when it is kept from him he vomits his food, and when had recourse to the symptom ceases. So at the present time it is the ordinary routine of his little sisters to collect mortar, which must not contain too much sand, as he is particular in the quality. Lime-water has been substituted, but this the child refuses to be contented with, and will have his food in a more solid form.

He is now suffering from small-pox, and, on waking up in the night, cries for a piece or two of lime before going to sleep again. The quanity consumed during twenty-four hours is rather more than half of a teacupful. His mother tells me he has never been weaned, and her custom has been to suckle her other children up to three years of age. To corroborate the mother's statement, I have frequently seen him partake of the mortar, which he crunches up and swallows with great relish.—*Med. and Surg. Reporter.*

---

MORTALITY OF INFANTS.—In an article on the above subject, Dr. J. H. Hanaford, writes the following regarding food given to infants. There is a possibility that the defective teeth seen in children is due to the improper starchy diet here mentioned.

"Many of our infants are sacrificed by improper feeding; the errors relating to times, to kinds of food given and to quantity. Most are fed too often, the ignorant mother supposing that the proper way of keeping the "wind out of the stomach" is by keeping that organ so gorged with food that no external "wind" can enter it, while the only "wind" to be feared is generated in the stomach, in consequence of excessive feeding. While some infants are fed but three times a day (the calf but twice,) I claim that, as soon as possible, or within a few days from birth, the three-hour system should be adopted—in ordinary cases—and strenuously followed, the time between meals to be granually increased with advance of age. No meals in the night, at least, after from four to six months. *Never* but one.

"Again many babes are brought to the table, eating about the same food taken by the family, which is as sensible as to provide them with the same clothing which is worn by adults. It is known by some, and should be by all medical advisers, and mothers, that the infant saliva, before the appearance of several teeth, is not provided with the necessary diastase for the digestion of starchy substances, without which starch cannot be properly digested and assimilated.

"And yet, babies are fed on pastry, rice, fine flour bread, arrowroot, corn starch, and the like, literally so starved as not to grow and be vigorous, like the babe properly cared for by an intelligent mothei. Some, at the suggestion of an ingorant medical attendant, are fed on a large quanity of water, combined with a little cream, a "vile compound" containing but little to nourish the babe, much less than *skimmed milk.* In this connection, it is proper to say that every true mother wishes to nurse her own child, this act constituting a very strong bond of sympathy and effection, besides being natural, and therefore healthful to the child.—*St. Louis Med. Journal.*

---

THE INCIPIENT STAGES OF INEBRIETY.—Dr. T. D. Crothers, whose facilities for experience ın all questions connected with alcoholism are very great, contributes a most readable and instructive article to the *Alienist and Neurologist* (April, 1885,) which he thus summarizes:

1. The study of inebriety reveals a well-marked disease passing through various stages, traceable by many and complex signs and symptoms.

2. The incipient stage seen before spirits are used is marked by dictetic delusions aud other symptoms of nerve and brain irritability, all of which seem to depend on heredity or some obscure injury to the nerve and brain centres.

3. A group of symptoms can be found in most cases that may be termed pathognomonic, and will be seen in the later stages fully developed.

4. These early symptoms appear after the first toxic use of alcohol, and in some cases go on to full development, or are held in abeyance by some unknown force.

5. Practically, the recognition and study of this stage opens up a field of prevention and cure that will attract great attention at an early day.—*Med. & Surg. Reporter.*

---

TEETH WITHOUT PLATES.—It is often very desirable to retain the roots of the natural teeth, specially if they are strong and in healthy condition, or if they can be made so, because there are a good many methods of inserting teeth by engrafting crowns on these roots and making them strong and perfect in every respect, without the annoyance of a movable plate. The resources of the skilful dentist are so numerous that he is not confined to a rut of experience and is not thwarted in his endeavors by a limitation of methods. Those who have made advancement their watchword have devised ways and means which are only to be made known to be appreciated. This advice then is to all—do not insist on methods which may be inferior, but leave the matter to the riper judgment of your dentist.—*Dental Guide*.

---

CALCIFICATION OF THE TEETH.—At any time prior to the "eruption" of the teeth, the enamel can be effected by hygienic measures, for good or for ill. And I feel sure that enamel may be improved in structure, even after twelve years of age, by proper regimen and diet. I *know* that the dentine may be. The already formed enamel is not as hard before birth as after more months of life.

If a child, at twelve years of age, has teeth poorly calcified, it, undoubtedly, has it bones in the same condition. That condition cannot be ascertained, as the bones are covered with soft tissues. The bones might be as poorly calcified as the poorly calcified teeth of a child, and yet be practically good for use, and grow no worse, while badly calcified teeth easily decay.

I have had a great deal of positive experience in improving the dentos of children from three to twenty years of age. I feel very certain that I have so instructed the expectant mother, as to cause the teeth of her child to be better than her own.

I improved the dentos of my own teeth after I was fifteen years old, by adopting hygienic habits; and I am sure that I have given my six children better dentos than their parents or grandparents had, by the same methods.

ST. LOUIS, MO. HENRY S. CHASE, M. D., D. D. S.

